# Diagnosing Late-Onset Psychosis in a Medically Complex Patient: A Diagnostic Challenge

**DOI:** 10.7759/cureus.62763

**Published:** 2024-06-20

**Authors:** Eduardo D Espiridion, HeeYun Na, Stacy Chou, Natasha Reddy

**Affiliations:** 1 Psychiatry, Drexel University College of Medicine, Philadelphia, USA; 2 Psychiatry, Reading Hospital, West Reading, USA; 3 Psychiatry, Drexel University College of Medicine, West Reading, USA

**Keywords:** meningioma, dural calcification, bipolar mania, delirium, psychosis

## Abstract

This case report explores various possible causes of late-onset psychosis and highlights the importance of follow-up care. We report the case of a 65-year-old female with minimal available medical history or contacts, who presented to the hospital after being found unconscious after three weeks of strange behaviors, including partition delusions, multiple phone calls and texts to her friend, and lack of sleep. In the following days, she had various symptoms consistent with delirium, psychosis, and mania. However, she was also found to have a dural calcification and urinary tract infection on imaging and laboratories, respectively. We attempted to distinguish these possible etiologies and understand the best course of action for such a patient with a limited medical history who was subsequently lost to medical follow-up. Utilizing the psychiatric interview, mental status examination, laboratory work, imaging, and available medical and psychiatric history can all help narrow down the most likely etiologies. However, the lack of data given during follow-up visits, regarding patient response to treatment, their full medical and psychiatry history, as well as their understanding of their diagnosis, poses a significant challenge in reaching a definitive diagnosis in such a patient. This underscores the critical need for follow-up care, especially for patients treated for psychosis in acute settings.

## Introduction

New-onset psychiatric symptoms in elderly patients are complicated to diagnose, especially if a patient has limited available medical and psychiatric history as well as undiagnosed comorbidities. Organic causes that must be ruled out, especially in the elderly, include substance use, metabolic syndromes, tumors, and infection [1]. Non-organic or psychiatric causes include bipolar disorder and schizophrenia. Physicians need to have a high index of suspicion in patients who present with atypical clinical features of a psychiatric disorder due to this wide array of potential causes. Atypical features of psychiatric disorders include focal neurological signs, the first episode outside the usual age of onset, or an unusual course of illness such as a single isolated episode with no subsequent symptoms, or poor response to adequate psychiatric treatment [2]. In such cases of atypical psychiatric symptoms, tests such as laboratories, neuroimaging, and cognitive impairment tests are essential to determine the origin of psychosis. An interaction between multiple causes is also possible in which it is important to distinguish and treat each contributing factor.

## Case presentation

A 65-year-old female presented to the emergency department (ED) of a community hospital at midnight after being found unconscious and unresponsive on her neighbor’s porch by a bystander. After receiving central noxious stimuli from the sternal rub on the ambulance, she became intermittently conscious and was able to follow some commands despite her altered mental status. She was awake but confused on arrival to the ED with a Glasgow Coma Scale score of 14, blood pressure of 202/101 mmHg, and heart rate of 84 beats/minute. The patient was somnolent and confused about the sequence of events, claiming she fell after tripping while walking her dogs, and awoke to people standing over her. Her symptoms were positive for retrograde amnesia, dizziness, and diplopia and were negative for headache, photophobia, nausea, and pain. Her strength and reflexes were intact, and the patient had no focal deficits or injuries.

The patient had a reported past medical history of pseudocholinesterase deficiency and vitamin D deficiency; however, she did not have a primary care provider (PCP) or pharmacy and took no medications other than a vitamin D supplement. She had no known family history of psychiatric illness. The patient endorsed no substance use other than occasional use of alcohol. She did not use alcohol in the days before this admission.

At the hospital, the patient underwent drug screening, urine culture, complete blood count, hepatic function, thyroid function, Lyme disease, and syphilis tests (Tables 1-3). The drug screen returned negative. Urinalysis was positive for leukocyte esterase, indicating a possible urinary tract infection (UTI). Her laboratory showed a sodium of 135 mEq/L (normal range: 135-145 mEq/L), glucose of 97 mg/dL, and elevated pH of 7.52 (normal range: 7.35-7.45) with increased oxygen and oxygen saturation as well as decreased carbon dioxide. The hemoglobin level was low at 11.9 g/dL. Her vitamin D and calcium were at the lower end of normal at 26 ng/mL (normal range: 20-50 ng/mL) and 8.7 mg/dL (normal range: 8.6-10.4), respectively. Electroencephalography and electrocardiography were not performed by the medical team at their discretion.

**Table 1:** Complete blood count with differential and complete metabolic panel.

Laboratory test	Result	Normal range
White blood cell count	8.1 × 10^3^ cells/mL	4.8–10.8 × 10^3^ cells/mL
Neutrophils	74.3%	40–75%
Lymphocytes	25%	20–45%
Red blood cell count	3.93 × 10^6^ cells/mL	4.00–5.40 × 10^6^ cells/mL
Hemoglobin	11.9 g/dL	12.0–16.0 g/dL
Hematocrit	36.1%	35.0–47.0%
Sodium	135 mEq/L	135–146 mEq/L
Potassium	3.5 mEq/L	3.5–5.3 mEq/L
Chloride	101 mEq/L	98–110 mEq/L
Calcium	8.7 mg/dL	8.6–10.4 mg/dL
Vitamin D 25-hydroxy	26.0 ng/mL	Deficient: <20 ng/mL
Glucose	97 mg/dL	65–99 mg/dL
Blood urea nitrogen	16 mg/dL	7–25 mg/dL
Creatinine	0.83 mg/dL	0.50–1.05 mg/dL

**Table 2:** Urinalysis results.

Laboratory test	Result	Normal range
Urine white blood cell count	34/HPF equivalent	0–5/HPF equivalent
Urine red blood cell count	3/HPF equivalent	0–5/HPF equivalent
Urine epithelial cells	5/HPF equivalent	0–4/HPF equivalent
Urine protein	10 mg/dL	Negative
Urine culture	Positive	Negative
Urine nitrite	Negative	Negative
Urine leukocyte esterase	Positive	Negative

**Table 3:** Venous blood gas test result.

Laboratory test	Result	Normal range
pH	7.52	7.31–7.41
pCO_2_	32 mmHg	41–51 mmHg
pO_2_	139 mmHg	25–40 mmHg
O_2_ saturation	99.0%	94.0–98.9%

**Figure 1:**
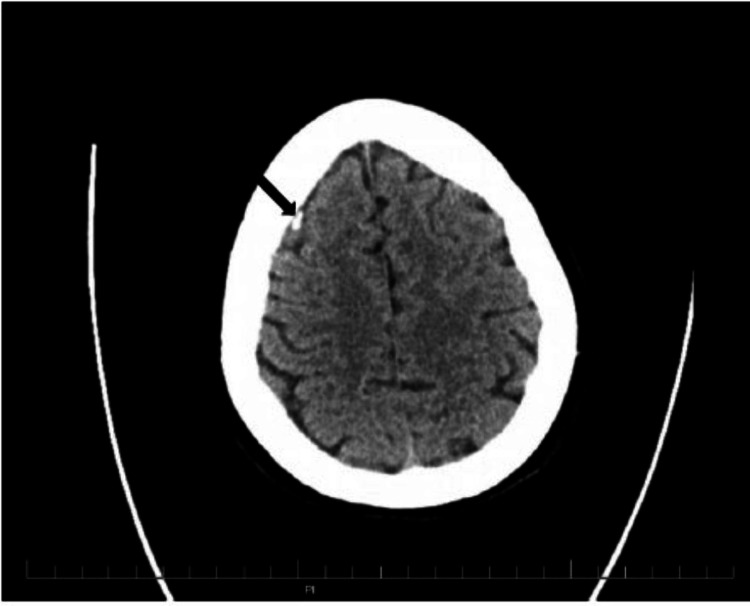
Head CT showing a 10 × 5 mm dense extra-axial calcification indicated with a black arrow.

On head/spine CT, the patient was found to have a 10 × 5 mm dense extra-axial calcification in the falx cerebri near the right frontal lobe. It was determined to be a large dural calcification or meningioma, as shown in Figure 1. The head CT was performed without contrast using dose reduction techniques, including automated exposure control, adjustment of mA and kV according to patient size, and use of iterative reconstruction technique. The spine showed multilevel degenerative changes with remote L1 compression deformity. On chest/abdomen CT, the patient had a 7 mm left pulmonary nodule at the lung base and an 11 mm hepatic lesion in the anterior right hepatic lobe.

While speaking on the phone with the patient’s long-term friend, she revealed that the patient had always been very isolated and had not seen a doctor in years, refusing to take medications. Although they had not spoken much over the past five years, the friend mentioned the patient being increasingly delusional, calling and texting her daily for the past three weeks. She had insisted she had cancer due to radon exposure in her home and had called 911 the previous night thinking someone had broken into her home. The friend also mentioned that the patient had a history of a psychotic break early in life during an abusive relationship.

Hours after being admitted to the medical floor by the trauma team, the patient became agitated, screaming and trying to hit the nurse. Despite being alert and oriented during a mini-mental status exam and scoring 27/30, and 10/64 on the Rivermead Post Concussion Symptoms Questionnaire, the patient exhibited concerning behavior. She gave partial or confusing answers to questions and had difficulty maintaining task awareness. She demonstrated disorganized speech, flight of ideas, with nonsensical profanity incorporated into her speech, and could not maintain a clear conversational path. The patient’s demeanor changed from being cooperative and pleasant at one point to expressing a flight of ideas, tangential speech, and paranoia. There was insomnia and the patient appeared to be responding to internal stimuli and tactile hallucinations. She yelled “stop touching me and leave me alone” while alone in her room.

Psychiatry was consulted the next morning for the altered mental status. During the interview, the patient was anxious due to difficulty processing questions, slapping herself when she could not organize her thoughts. She was hypervigilant and reported not sleeping the past couple of nights, as well as not sleeping or eating in the days before arriving at the hospital. On the mental status exam, her appearance was disheveled, and she was dressed in a hospital gown. Her behavior was guarded with intermittent eye contact and psychomotor agitation. Her speech was pressured, her mood was irritable, and her affect was labile. Her thought processes were concrete, and her thought content consisted of paranoid delusions, delusions of grandiosity, tactile hallucinations, and flight of ideas, although she denied any suicidal or homicidal ideations, intent, or plans. She had impaired judgment and poor insight, as well as impaired concentration and memory. The patient was alert and oriented with appropriate language and adequate fund of knowledge.

The patient was diagnosed with a major neurocognitive disorder and unspecified psychosis. She was started on olanzapine 5 mg nightly and required intramuscular olanzapine after becoming agitated and aggressive again. The medicine service ruled out concussion and alcohol and drug use, and she was given ceftriaxone for a possible UTI. When the psychiatry team saw her again the next day, she was alert and oriented, claiming she was aware of her agitated state and rationalizing that she was upset because she “wants to leave.” She had poor frustration tolerance, slightly pressured speech, paranoia, and poor concentration but her delirium was improved.

The patient was hospitalized for five days. On the last day, she appeared well groomed with normal speech, euthymic mood, constricted affect, normal thought process and content, and fair insight and judgment. She was calmer and more cooperative, alert, and oriented with improved concentration and attention span. She denied any manic or psychotic symptoms. Antibiotics and olanzapine were continued. Despite her improvement in symptoms, the medicine and psychiatry services were unable to conclude the definitive etiology of her psychosis. Her UTI, large dural calcification on imaging, hepatic and pulmonic lesions, hyponatremia, malnutrition, and sleep deprivation suggested an organic cause, while her history of previous psychiatric hospitalization and symptoms of delirium, mania, and psychosis suggested a psychiatric component as well. Her limited available medical, psychiatric, and family history made her case even more complex.

The patient was determined to have the capacity to sign herself out that day and was recommended to follow up with psychiatry as an outpatient and to follow up for brain MRI for calcification, chest CT for pulmonary nodule, and liver MRI for hepatic lesion. However, while the patient was contacted numerous times to complete a follow-up, a follow-up appointment for these issues did not occur. Approximately a week after discharge, the patient attended an outpatient internal medicine visit but never followed up with psychiatry.

## Discussion

Separating the complex interplay of factors in this patient is a difficult task, but using her laboratory results and imaging, the possible organic causes can be investigated. Upon admission, the patient was diagnosed with a UTI based on elevated leukocyte esterase in her urinalysis and was given antibiotics. UTI can both precipitate or exacerbate neuropsychiatric disorders and the resolution of psychiatric symptoms upon beginning antibiotics is the main confirmation [3]. Her condition did improve within a couple of days of the antibiotics, but because the olanzapine was started around the same time, it is difficult to distinguish the effect of each. Her acute presentation, age, and delirium align with UTI being the cause of her symptoms. In addition, the patient’s delusions beginning three weeks earlier would serve as an exacerbating factor. UTI generally causes symptoms of delirium more than psychosis as well, so this could explain a picture of acute delirium that may have presented in addition to other underlying psychiatric issues.

The large calcification in the patient’s frontal lobe, suspected to be a meningioma but requiring further imaging, is another major organic cause to consider. Meningiomas are the most common primary tumors of the brain and frontal meningiomas are known for their late onset, presenting only with psychiatric symptoms [4,5]. Mania, psychosis, and visual hallucinations are the most common manifestations and are mostly associated with right-sided lesions [6,7]. Symptoms usually resolve with resection of the mass as well [6]. Mania secondary to brain lesions, especially affecting frontal, temporal, and subcortical limbic brain areas, should be considered in patients with a history atypical for classic bipolar and first manic episode after age 40 [2]. One case study discusses a 73-year-old woman with a four-week history of mania, a nine-month history of delusions, and no past psychiatric history who was found to have a 5 × 5 cm frontal mass and no cortical atrophy, similar to our case [8]. However, our patient had a much smaller calcification and appeared to have a more abrupt onset of symptoms. Most meningiomas 2.5 cm or less in diameter do not proceed to cause symptoms in the five years following their discovery [9], making this patient’s imaging findings a less likely cause of her symptoms. However, it is also important to consider that our patient lived alone with no close contacts to attest to whether she was having symptoms in the months before her hospitalization. Because 78% of patients with brain tumors develop psychiatric symptoms and 19% develop hallucinations, neuroimaging is a critical component in diagnosing atypical psychosis [6]. Yet, given the fact that the patient responded to olanzapine which improved her psychosis, this puts mania secondary to meningioma less likely on the differential.

Based on her reported past medical history of vitamin D deficiency, acute psychosis secondary to vitamin D deficiency was also considered. For instance, past literature has reported that patients with vitamin D deficiency were 3.5 times more likely to experience hallucinations, paranoia, or delusions [10], and patients with first-episode psychosis have significantly lower concentrations of vitamin D [11]. Although our patient had a past medical history of vitamin D deficiency, her laboratory vitamin D values at the time of admission did not meet the criteria for vitamin D deficiency, although it did fall at the lower end of the normal range.

The patient also had a reported history of anemia, which could be a precipitating factor for her delirium. The patient presented with a hemoglobin level of 11.9 g/dL at the time of admission, and she also reported a history of anemia as a child. Anemia has been shown to be associated with an increased risk of delirium in older patients, especially in postoperative settings [12]. Studies indicate that anemia can contribute to cerebral hypoxia, which may precipitate delirium, particularly in vulnerable populations such as the elderly [13]. Moreover, the patient may have had a fall due to syncope secondary to anemic shock as she was found unresponsive on her neighbor’s porch. Malnutrition, dehydration, and sleep deprivation in the days before admission were likely exacerbating factors, but maybe not causative factors as she had already been experiencing two weeks of psychiatric symptoms by then. As many organic and non-organic causes can lead to acute delirium in patients, especially in the elderly, delirium is a likely diagnosis. However, the etiology remains unknown to pinpoint a single cause of delirium and the aforementioned factors are more likely exacerbating factors for the patient’s delirium. Some other organic factors that were considered included neurological damage or concussion due to the fall; however, this was ruled out based on imaging.

The main component of this patient’s picture that points to a psychiatric etiology is her prior history of psychiatric hospitalization. Unfortunately, the lack of records from that incident, the lack of family and friends to obtain the story from, and the patient’s inaccurate storytelling make it challenging to rule out organic versus non-organic causes of her psychosis.

The patient exhibited many qualities suggesting a diagnosis of delirium such as waxing and waning symptoms and sundowning. Her delirium could also be a component of a more complex diagnosis of delirious mania, which involves the rapid onset of delirium, mania, and psychosis, not associated with prior toxicity, physical illness, or mental disorder. This was seen in a case report of a 60-year-old female with a week of mania, delirium, inappropriate laughter, hypersexuality, and no significant psychiatric history [14]. Our patient had all of the above symptoms as well and shared similarities to this case including age and sex.

Another possible psychiatric diagnosis is very late-onset schizophrenia-like psychosis (VLOSLP). VLOSLP has an age of onset over 60 in the absence of mood disorder or neurological illness [15]. It is more common in females but is a rare condition overall. It consists of psychotic symptoms such as hallucinations, paranoid and partition delusions, and fewer negative symptoms such as affect flattening [15]. Partition delusions are an interesting case in which structures that normally act as barriers to movement, sight, and sound (like walls, floors, ceilings, and doors) become permeable [16]. This is consistent with the patient’s positive psychotic symptoms, and with her paranoid and partition delusions, convinced that radon or her neighbor’s marijuana was leaking through the basement and vents. Her age at onset makes this diagnosis possible and would be deemed more plausible if organic causes were ruled out. Furthermore, the lack of abnormal neurological signs and cognitive impairment noted during the exam and the patient’s improvement of psychosis with olanzapine make this diagnosis likely.

While this was a complicated patient to treat due to her multiple comorbidities, her lack of close contacts and doctor visits, and her unavailable medical history, comparing the slightly varying psychiatric presentations of each diagnosis can help physicians narrow down her disease process. In this case, antibiotics may have helped treat her delirium secondary to UTI, and olanzapine may have helped treat her mania and psychosis which could have been secondary to meningioma, or VLOSLP. There is also a possibility of post-ictal psychosis but the patient did not stay in the hospital long enough nor she followed up in the outpatient to verify this.

In addition, this patient was largely lost to follow-up, which hindered the ability to concretely determine the cause of her altered mental status. Her PCP note from a few weeks following her hospitalization stated that the patient was feeling “woozy, light-headed, and with brain fog,” and that she was interested in weaning off her olanzapine. The encounter notes also mentioned a psychiatric appointment in the weeks following; however, the patient did not follow through on this appointment. The difficulty of following up on this patient in psychiatry but not within primary care indicates that having an integrated behavioral health (BH) and primary care service could have enabled a more seamless handoff of patient care. BH and PCPs working in tandem, within the same facility and using the same electronic medical record, can communicate in person, discuss cases, and otherwise collaborate. This case further emphasizes the benefits of an integrated care model that can provide warm hand-offs between the PCP and BH provider should a need be identified, thereby reducing potential barriers to care. If such a thing were available, perhaps this patient’s uncertain diagnosis could be fully differentiated.

The benefits of integrated care are well-documented. Research shows that integrating BH into primary care settings significantly improves patient engagement and follow-up rates for psychiatric appointments [17]. Had she been part of an integrated care system, the likelihood of her attending follow-up appointments could have been higher, facilitating further imaging of her brain, liver, and lung lesions to determine whether she had subsequent mood symptoms or more psychotic episodes. An integrated care model could have provided the necessary support and coordination to ensure that the patient received comprehensive follow-up care, thereby reducing potential barriers to care, and improving her overall health outcomes. This approach emphasizes the importance of integrated BH and primary care services in managing complex cases like this one, where the interplay between medical and psychiatric factors is significant.

## Conclusions

In a patient with late-onset symptoms of psychosis, mania, and delirium, there are multiple differential diagnoses to consider, including those of both organic and psychiatric nature. These possibilities need to be carefully considered and assessed using laboratory, imaging, mental status exams, and clinical judgment. It is crucial to understand the various etiologies of these symptoms and rule out medical causes, especially in elderly patients in whom many metabolic, drug-induced, and infectious factors can have psychiatric effects. Moreover, understanding the subtle variations in the presentation of symptoms, patient population, and interaction between organic and non-organic factors can help deduce the etiologies of such symptoms. However, for patients with a limited medical and psychiatric history, close follow-up care can result in more effective and accurate treatment for the patient.

## References

[1] Joyce EM (2018). Organic psychosis: the pathobiology and treatment of delusions. CNS Neurosci Ther.

[2] Satzer D, Bond DJ (2016). Mania secondary to focal brain lesions: implications for understanding the functional neuroanatomy of bipolar disorder. Bipolar Disord.

[3] Chae JH, Miller BJ (2015). Beyond urinary tract infections (UTIs) and delirium: a systematic review of UTIs and neuropsychiatric disorders. J Psychiatr Pract.

[4] Chaari B, Nefzi R, Mhedhbi N, Khelifa E, Aissa A, El Hechmi Z (2017). Frontal meningioma and bipolar disorder: etiopathogenic link or co-morbidity? A case report. Eur Psychiatry.

[5] Aparicio L, Perico C, de Andrade A (2011). Mania secondary to meningioma. Int Clin Psychopharmacol.

[6] Shen SQ, Sinha N, Perrin RJ, Ghoshal N, Hawasli AH, Womer FY (2018). Acquired mania associated with a left temporal meningioma. Bipolar Disord.

[7] Mithun S, Hegde D, Sreedaran P (2017). Frontal lobe meningioma presenting as mania. Arch Ment Health.

[8] Byrne A, Henry S (2020). Meningioma and psychosis - cause or coincidence?. Prog Neurol Psychiatry.

[9] Sughrue ME, Rutkowski MJ, Aranda D, Barani IJ, McDermott MW, Parsa AT (2010). Treatment decision making based on the published natural history and growth rate of small meningiomas. J Neurosurg.

[10] Gracious BL, Finucane TL, Friedman-Campbell M, Messing S, Parkhurst MN (2012). Vitamin D deficiency and psychotic features in mentally ill adolescents: a cross-sectional study. BMC Psychiatry.

[11] Crews M, Lally J, Gardner-Sood P (2013). Vitamin D deficiency in first episode psychosis: a case-control study. Schizophr Res.

[12] Al Farsi RS, Al Alawi AM, Al Huraizi AR, Al-Saadi T, Al-Hamadani N, Al Zeedy K, Al-Maqbali JS (2023). Delirium in medically hospitalized patients: prevalence, recognition and risk factors: a prospective cohort study. J Clin Med.

[13] Joosten E, Lemiengre J, Nelis T, Verbeke G, Milisen K (2006). Is anaemia a risk factor for delirium in an acute geriatric population?. Gerontology.

[14] Jacobowski NL, Heckers S, Bobo WV (2013). Delirious mania: detection, diagnosis, and clinical management in the acute setting. J Psychiatr Pract.

[15] Van Assche L, Morrens M, Luyten P, Van de Ven L, Vandenbulcke M (2017). The neuropsychology and neurobiology of late-onset schizophrenia and very-late-onset schizophrenia-like psychosis: a critical review. Neurosci Biobehav Rev.

[16] Cort E, Meehan J, Reeves S, Howard R (2018). Very late-onset schizophrenia-like psychosis: a clinical update. J Psychosoc Nurs Ment Health Serv.

[17] Filippi MK, Waxmonsky JA, Williams MD, Robertson E, Doubeni C, Hester CM, Nederveld A (2024). Integrated behavioral health implementation and training in primary care: a practice-based research network study. J Am Board Fam Med.

